# Department of Therapeutics

**Published:** 1893-08

**Authors:** David Cerna

**Affiliations:** Demonstrator of Physiology in the Medical Department of the University of Texas, Etc.; Galveston


### DEPARTMENT OF THERAPEUTICS.

[Under the charge of David Cerna, M. D., Ph. D., Demonstrator of Physiology in the Medical Department of the University of Texas, etc.]

THE THERAPEUTIC APPLICATIONS OF KOLA-NUT.—John V. Shoemaker publishes an interesting article on the therapeutic actions of kola-nut (*American Med.-Surg. Bulletin*, May, 1893). The drug is really a *seed*, and is the product of the *Sterculia acuminata*, indigenous to Africa. It contains about 2.3 per cent. of an alkaloid analogous to theine and caffeine, 0.023 per cent. of thebromine, together with tannic acid, sugar, albumin, cellulose, starch, fat, and fixed salts. The drug is said to increase the appetite and facillitate digestion. According to the studies of Shoemaker it has been found of service in a large variety of disorders characterized by debility. He has employed it, with apparent success, in migrane, simple anæmia, melancholia, ulnar neuritis, vaso-motor neuroses, gastro-intestinal irritation, irregular heart, pulmonary tuberculosis, tubercular diarrhœa, dyspepsia, gastro-enteritis; in the convalescence from severe ailments, such as typhoid fever, acute pneumonia, rheumatism, influenza, etc.; in catarrhal jaundice, bilious diarrhœa, and finally, in cases of prolonged suppuration from diverse causes. The powder of the drug may be administered in doses of from five grains to one and even two drachms, given preferably in capsules, or compressed tablets or lozenges. The author has found the remedy, on the whole, *an excellent reconstituent tonic.* It is readily taken, and acceptable to the stomach; takes the place, to a certain extent, of alcoholic stimulant, and has a more permanent effect than alcohol.

THE USES OF THILANIN—In an interesting paper, George Henry Fox (*Amer. Med.-Surg. Bulletin*, May, 1893) published his experience with the use of thilanin in the treatment of skin diseases, both in private and hospital practice. The author's estimate of the drug is not particularly favorable. He has failed to find any justification of the claims which have been made in its behalf. From the good results, however, which followed its use in the cases of erythematous lupus and seborrhœa, he appears to be inclined to give the remedy a further trial in similar cases; but even here would not recommend it as possessing any *exceptional* virtue. Some of the cases, taken alone, might serve as a basis for the highest praise of thilanin, while others would seem to justify its utter condemnation. Taking all together, they appear to prove that the drug in question, like most of the "new remedies," is of some value under certain conditions, but is far from being the valuable addition to our dermatological pharmacopœia, which the paper of Saalfeld would lead one to suspect.

GALLANOL IN THE TREATMENT OF PSORIASIS AND ECZEMA.—At the recent annual meeting of the French Society of Dermatology and Syphilography, according to the report in the *Progres Medical*, for April 22, Dr. Cazeneuve and Dr. E. Rollet, of Lyons, made known the results obtained in the treatment of psoriasis and eczema by means of gallanol. This body, isolated by them in a state of purity from gallic acid, acts powerfully upon the skin as a reducing agent. In psoriasis the affected part is painted with a ten per cent. solution and the layer is covered with traumaticine. After a very short time the psoriasis is found to have disappeared. In chronic eczema, a ten per cent. or twenty-five per cent. ointment causes the itching to subside and brings about a prompt cure.—*N. Y. Medical Journal*, May 13, 1893.

TOLERANCE TO NITRO-GLYCERINE.—A curious instance of unusual acquired tolerance to nitro-glycerine is reported by Geo. Evans Reading, of Woodbury, N. Y. (*Therapeutic Gazette*, May 15, 1893). The case was that of a woman, fifty-seven years of age, in whom a chronic interstitial nephritis had been diagnosed. She was placed on nitro-glycerine in 1.100 of a grain doses, in solution, before meals, and on chloride of gold and sodium, in 1.20 of a grain doses, after meals. The nitro-glycerine produced from the first its physiological effects, but these soon wore off, and the dose was gradually increased. The increase of the dose was steadily continued, until in less than one year from the time of beginning the patient was taking a teaspoonful of a ten per cent. solution at each dose, this being equal to six grains of the pure drug. This dosage was maintained without any further increase for several weeks. The effect upon the kidneys was marvelous. From five quarts per diem the urine decreased to four pints, and from a sp. gr. of 1002, with no color and not even a urinary odor, it increased to a sp. gr. of 1018, and presented then a natural color. The disagreeable symptoms exhibited by the patient, such as vertigo and indigestion, were considerably ameliorated, and, indeed, apparently cured. The chloride of gold and sodium was increased to 1.10 of a grain. This remarkable case of tolerance to nitro-glycerine is put on record by the author to emphasize the fact that to secure the good effects of which the drug is capable in the disease in question, it must be pushed to the utmost tolerance of the patient, whatever that dosage may be.

WHAT BENEFIT CAN EAR-PATIENTS DERIVE FROM NASAL TREATMENT.—Under the above title H. Gradle, of Chicago (*The Journal of the American Medical Association*, June 3, 1893), publishes an interesting commnnication. A review of his observations from the aural point of view leads the author to the following conclusions:

1. Acute suppurative inflammation of the middle ear, if not treated, has a tendency to become chronic, the tendency increasing with the age of the patient.

2. Chronic suppuration of the middle ear rarely heals without treatment. Neither acute nor chronic purulent otitis is influenced by nasal treatment, but the liabiltity of relapses after their cure is decidedly lessened by the removal of naso-pharyngeal anomalies.

3. Acute catarrh of the middle ear will generally terminate in complete recovery under aural treatment, and sometimes even without it, provided there are no persistent nasal or pharyngeal lesions. But when these are present the disease is more likely to become chronic in spite of aural treatment, and in many instances can either not be cured or if improved will speedily relapse unless the normal state of the nose and throat is restored.

4. Proliferating or adhesive disease of the middle ear is the consequence of retro-nasal catarrh and its course is determined by the course of the disorder causing it. Aural treatment alone is practically useless in this form of trouble, while nasal treatment, if successful as far as the catarrh is concerned, will also arrest the ear disease. The restitution of hearing, however, depends upon the length of time the disease has lasted and is often aided by ear treatment after the cure of the retro-nasal catarrh.

